# Cytotoxic effects of dose dependent inorganic magnesium oxide nanoparticles on the reproductive organs of rats

**DOI:** 10.1080/07853890.2023.2258917

**Published:** 2023-09-28

**Authors:** Ghada H. Naguib, Gamal S. Abd El-Aziz, Rayyan A. Kayal, Abdulghani I. Mira, Maher S. Hajjaj, Hisham A. Mously, Mohamed T. Hamed

**Affiliations:** a Department of Restorative Dentistry, Faculty of Dentistry, King Abdulaziz University, Jeddah, Saudi Arabia; b Department of Oral Biology, Cairo University School of Dentistry, Cairo, Egypt; c Department of Anatomy, Faculty of Medicine, King Abdulaziz University, Jeddah, Saudi Arabia; d Department of Oral Basic and Clinical Sciences, Faculty of Dentistry, King Abdulaziz University, Jeddah, Saudi Arabia; e Department of Oral and Maxillofacial Prosthodontics, Faculty of Dentistry, King Abdulaziz University, Jeddah, Saudi Arabia; f Department of Fixed Prosthodontics, Cairo University School of Dentistry, Cairo, Egypt

**Keywords:** Magnesium oxide nanoparticles, testis, ovary, sex hormones, oxidative stress, rats

## Abstract

**Introduction:**

Magnesium oxide nanoparticles (MgO NPs) have a variety of applications that have contributed to their elevated popularity, however, the safety and toxic effects on humans are also of concern with these increased applications. There is insufficient data regarding the effect of MgO NPs on reproductive organs, which are crucial aspects to the body’s vital physiological functions. The present study was undertaken in male and female rats to assess the reproductive toxicological potential of two doses (low versus high) of MgO NPs on testicular and ovarian tissues. The toxicity was evaluated using histological, hormonal, and oxidative parameters.

**Material and Methods:**

In this work, magnesium oxide nanoparticles (MgO NPs) were synthesized by the sol-gel route and were characterized by X ray diffraction analysis (XRD) and Fourier transform infra-red spectroscopy (FTIR). Forty-eight adult Wister albino rats were used in this experiment which were divided into groups of male and female, and then further into control, low dose MgO NPs, and high dose MgO NPs. The low dose used was 131.5 mg/kg b.w. (1/10 LD50) while the high dose used was 263 mg/kg b.w. (1/5 LD50). All doses were given orally by gastric tube. After 4 weeks, blood samples were collected to investigate the level of sex hormones and both ovarian and testicular tissues were examined for variable oxidative parameters and histopathological changes by light microscopy.

**Results:**

The obtained findings showed that high dose of MgO NPs produced considerable changes in sex hormones and stress parameters in both male and female rats in comparison to the low dose and control groups. Histomorphometric analysis demonstrated the presence of histopathological alterations in the testicular and ovarian tissues.

**Conclusion:**

The results of this study showed dose-dependent adverse effects of MgO NPs on the testis and ovary both functionally and histopathologically as compared to the control rats.

## 1. Introduction

With the consistently expanding growth and development in the field of nanotechnology, the production and application of many types of nanoparticles (NPs) have become more prevalent in various aspects of daily activity. NPs are beneficial for human life due to their interesting properties and easy usability where they exhibit a wide variety of physico-chemical properties, which have implications on their absorption and distribution to the human body’s organs as well as their metabolism and excretion **[**1–3]. Given these unique properties, NPs have been showing promising and large-scale applications in several fields such as cosmetics **[**4], agriculture **[**5], biosensors and biotechnology **[**6], cancer treatment [7] dentistry **[**8], and drug delivery **[**9]. However, due to the widespread applications of these NPs in various aspects, human exposure is inevitable, which raises questions about their potential health hazards **[**10].

In this respect, the toxic effects of some NPs were demonstrated in several previous studies where it was documented that various forms of NPs have been proven to be able to cross biological barriers and membranes, causing some damaging adverse effects on vital organs such as the brain, liver, kidney, and lung, which are the most investigated target organs **[**11–14].

In addition, the reproductive toxicity of NPs has just lately received attention. This system represents a delicate biological process essential for the transferring of genetic information to offspring and is accordingly profoundly influenced by any exogenous hazard that can occur **[**15]. There is evidence that some NPs can pass from the reproductive barriers systems such as the blood-testis barrier, placental barrier, and epithelial barrier, and then accumulate in the testis, epididymis, ovary, and uterus and eventually cause damage to these organs **[**16,17]. The accumulation of NPs in the reproductive system can have adverse effects on sperm, which can include alterations of quantity, quality, motility, and sperm morphology. The detrimental effects on the oocytes involve the development of primary and secondary follicles, the number of mature oocytes and their reduction, and the levels of secreted hormones, and can manifest in changes in sexual behaviour **[**18].

Of all NPs, metal oxide NPs such as titanium dioxide (TiO2), zinc oxide (ZnO), iron oxide (Fe2O3), and copper oxide (CuO), have received heightened attention due to characteristics obtained from the NPs’ high surface-to-volume ratio. These metal oxide NPs have been implemented into biomedical applications as used in targeted drug delivery, cancer therapy and treatment, gene therapy, DNA examination and analysis, antibacterial agents, biosensors, and magnetic resonance imaging **[**19,20]. However, these NPs can be released into the environment through manufacturing processes, leading to possible human exposure, which may potentially cause cytotoxicity due to the ability of metal oxide nanoparticles to generate reactive oxygen species (ROS), inflammation, and interfere with mitochondrial function **[**21,22].

Among the metal oxide NPs is magnesium oxide (MgO) NPs, which has garnered broad scientific consideration due to its unique properties, as it is a simply synthesized and chemically stable compound, in addition to its biocompatibility, biodegradability and low cost **[**23**]**. MgO NPs are globally used in multiple medical and therapeutic fields, with some uses involving being utilized as an antacid, as well as in detoxifying preparation due to their significant antibacterial activity against gram-positive and gram-negative bacteria, and food-borne pathogens such as E. coli and Salmonella Enteritidis **[**24–26]. Furthermore, these properties also allow for clinical use in dentistry as parts of polymeric filling, restoration materials, and barrier membranes **[**8,27–29]. They also present with good anti-arthritic and anti-cancer activity **[**24,25,30]. However, with the increased application of MgO NPs, there is a concerning lack of knowledge related to their impact on human health.

To our knowledge, no previous research has been conducted to investigate the toxic profile of MgO NPs on reproductive health in either males or females. Therefore, the current study was undertaken in male and female rats to assess the toxicological reproductive potential of two doses (low versus high) of MgO NPs on both testicular and ovarian tissues. This toxicity was evaluated using histological, hormonal, and oxidative stress parameters.

## 2. Material and methods

### 2.1. Chemicals

MgO NPs (MgO < 50 nm, 99.9%, CAS No. 1309-48-4) were purchased from Sigma Chemical Co. Ltd (St Louis, MO, USA). The Elisa kits for FSH (Cat No. 10001), LH (Cat No. 10004), Testosterone (Cat No. 10007), Oestradiol (E2) (Cat No. 10009) and Progesterone (ELISA, (Cat No. 10005) were purchased from PerkinElmer Company, Hayward, California, USA. Additionally, the Lipid Peroxidation (MDA) Assay Kit (ab118970), Superoxide Dismutase (SOD) Activity Assay Kit (ab65354), Catalase (CAT) Activity Assay Kit (ab83464), and Glutathione Peroxidase (GPx) Assay Kit (ab102530) were purchased from Abcam, USA.

### 2.2. Preparation of MgO NPs solution

The MgO NPs were prepared by sol-gel method **[**31]. The stock solution (1 mg/ml) was made freshly before each new experiment by suspending MgO nanopowder in 0.9% sodium chloride (dissolved in Millipore filtered water). The resulting solution was stirred overnight and finally ultra-sonicated using an ultrasonic bath (30.8 W; Elma Schmidbauer GmbH, Germany) for 30 min **[**32].

### 2.3. Characterisation of the MgO NPs

#### 2.3.1. X Ray diffraction analysis (XRD)

XRD measurements of the MgO NPs were performed using a Shimadzu XRD-6000X-ray powder diffractometer using Cu Kα radiation (0.15406 nm at 15 kV and 30 mA for the X-ray tube). For every measurement, the phase type and content were analyzed at 5° min-1. A complete 2θ scan was performed between 10° and 80° **[**33].

#### 2.3.2. Fourier transform infra-red spectroscopy (FTIR)

FTIR spectra of MgO NPs were obtained utilizing the KBr pellet method using a Nicolet spectrophotometer with a DTGS TEC detector enabled. The spectra were recorded from 4000 to400cm-1 **[**34].

### 2.4. Animals

Forty-eight sexually mature Sprague Dawley rats (24 males and 24 females) (weighing between 180 – 200 gm) were used in this study. They were acquired from the Animal Research Unit, Faculty of Pharmacy, King Abdulaziz University, Saudi Arabia. Before the experiment was carried out, the rats resided in an isolated animal room for a week. During this period, the conditions were strictly controlled at 12-h light/dark cycle, with a constant temperature of *24 °C* ± 2 °C, and a relative humidity of 50%±10%. The rats were allowed to feed on standard food of Purina rat chow and water ad-libitum. All animal procedures were conducted according to the guidelines of the Faculty of Pharmacy’s Research Ethics Committee, King Abdulaziz University (# PH-1442-63). Handling of animals was in strict accordance with the ARRIVE guidelines.

After two weeks acclimatization in laboratory condition, both male and female rats were divided into 3 groups (*n* = 8):

- Control group: given distilled water orally.
- Low dose treated group: received low dose (131.5 mg/kg bw equal to 1/10 LD50) of MgO NPs orally once/day **[**35].
- High dose treated group: received high dose (263 mg/kg bw equal to 1/5 LD50) of MgO NPs orally once/day **[**35].

### 2.5. Treatment and collection of samples

All rats’ treatments began at 10 o’clock in the morning and administered through a gastric tube. Four weeks after the start of the treatment, along with overnight fasting, blood samples from different rats were withdrawn from retro-orbital venous plexus by capillary tubes under ether anaesthesia, centrifuged at 3000 rpm to separate the serum that was stored at −80 °C until further analyses. Then the male and female rats were euthanized by decapitation. The abdomen was opened longitudinally by a lower median incision to extract both right and left testes and ovaries, which were immediately washed with ice-cold PBS. The right testes and ovaries were cut transversely and preserved in 10% neutral buffer formalin (NBF) for subsequent histological studies, while the left testes and ovaries were immediately wrapped in aluminium foil and frozen in liquid nitrogen to be stored at −80 °C.

### 2.6. Sex hormone Assays

All hormones were assayed using specific enzyme-linked immunosorbent assay (ELISA) commercial kits according to the manufacturers’ protocol (DRG Instruments GmbH, Marburg, Germany). The serum level of testosterone was measured as a marker for male reproductive functions, while the serum levels of follicle-stimulating hormone (FSH), luteinizing hormone (LH), progesterone (P), and oestradiol (E2) were determined as markers for female reproductive functions. All the samples were analyzed in the same assay to avoid inter-assay variability.

### 2.7. Gonads homogenates and Assay of lipid peroxidation and antioxidant markers

Frozen pieces of testis and ovary were minced and homogenized using a homogenizer (Cole‑Parmer Inst. Co., USA) with buffer (0.1 M phosphate buffer saline + 1% Triton ×100, pH 7.4). The homogenates were centrifuged at 4000× g at 4 ◦C for 20 min and the clear supernatant was frozen and held at −80 ◦C until further processing. The levels of lipid peroxidation indicators and antioxidative enzyme activity in the supernatants were evaluated using a spectrophotometer and commercial Biodiagnostic kits (Cell Biolabs Inc., USA). The lipid peroxide malondialdehyde (MDA) level was measured according to the method used before by [36]. The testicular MDA concentration was calculated as nmoL/g tissue. The superoxide dismutase enzyme (SOD) activity was measured according to [37], the testicular SOD activity was quantified as U/mg tissue. The catalase enzyme (CAT) activity was measured in the supernatant according to (38]. The result of CAT activity was expressed in μmol/mg tissue. The peroxidase enzyme (GPx) was assessed according to the method previously used by [39]. The result of GPx activity was expressed in μg/mg tissue.

### 2.8. Histopathological study

The NBF fixed pieces of both testis and ovary were processed through ascending grades of alcohol then cleared with xylene and embedded in paraffin blocks using the automatic processor of the histopathology lab. Serial 5 *µ*m slices were prepped and stained using haematoxylin and eosin (H & E) to evaluate the general structure **[**40]. The slides were blindly examined under the light microscope. Photography of the slides from different groups at various magnifications was obtained using a camera-loaded microscope Olympus BX53 (Olympus, Tokyo, Japan). All the procedures of tissue processing, photography, and histomorphometric measurements were carried out at the Department of Anatomy, Faculty of Medicine, King Abdulaziz University.

### 2.9. Histometric analysis

The histometric measurements were performed using the image analyzer Image J software. For this analysis, 6 non-overlapping fields of each testis and ovary section (X 200) were selected randomly and recorded. For each specimen, the mean values and standard deviations (SD) were calculated. For the testis, the following parameters were assessed as described previously by [41]:

1. The seminiferous tubule diameter (STD): measured across the minor and major axes of the seminiferous tubule.
2. The seminiferous germinal height (SGH): measured as the space between the basement membrane and the edge of the lumen of the seminiferous tubule.

For the ovary, the number of ovarian follicles/fields including primordial, primary, preantral, antral follicles, corpora lutea, and atretic follicles was calculated. The ovarian follicles were classified according to the method of (42] where the primordial follicles contained an oocyte surrounded by a partial or complete layer of flat cells, and the primary follicles showed a single layer of cuboidal cells. Preantral follicles showed 2-4 layers of cells with no antral space. Antral follicles contain 3 or more layers of cells and a clearly defined antral space. Atretic follicles appeared irregular and shrunken with degenerated cells.

### 2.10. Statistical analysis

All obtained measurements were expressed as means ± standard deviation (mean ± SD). The data analysis was completed with the Statistical Package for Social Science (SPSS) Version 16 for Windows. Comparison of variables between groups was performed using one-way analysis of variance (ANOVA) followed by Tukey’s post hoc test. Statistical significances were considered at *p*-value < 0.05.

## 3. Results

### 3.1. Characterization of the MgO NPs

#### 3.1.1. X-ray diffraction (XRD)

**Figure 1.**
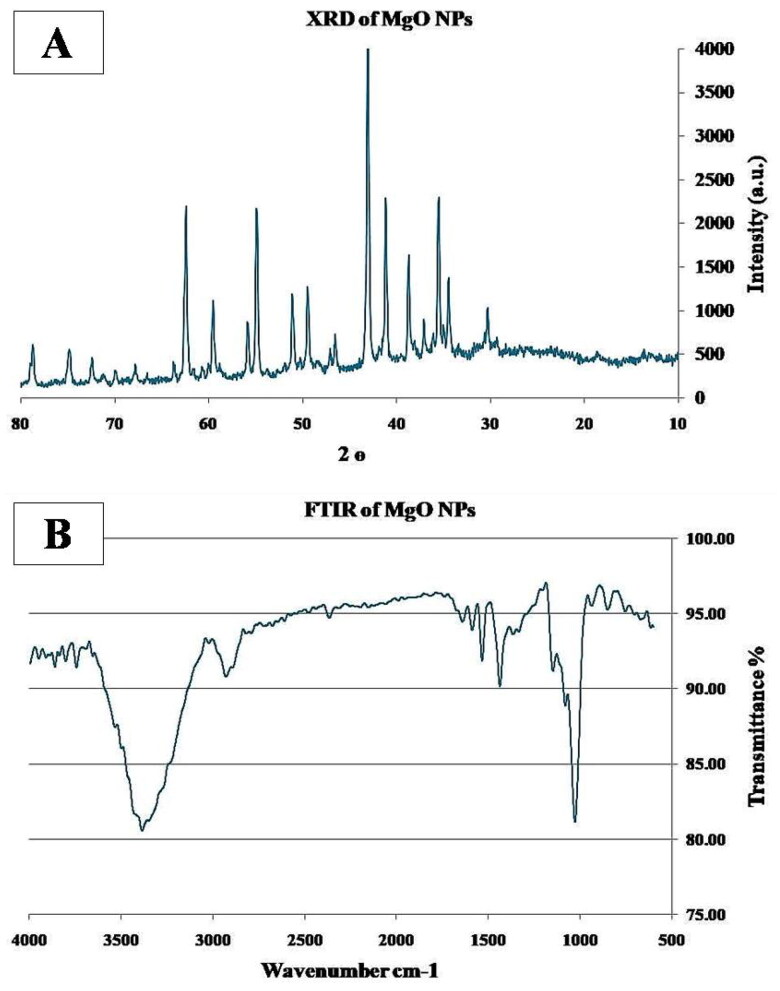
Showing the Characterization of MgO NPs A) XRD, B) FTIR.

The high-intensity and sharp peaks in the XRD spectra demonstrate that MgO is highly crystalline in nature. MgO shows its respective peaks at 36.98◦, 42.95°, 62.40°, 74.80°, and 78.75◦ 2θ due to the (111), (200), (220), (311), and (222) planes, respectively, which confirms the presence of cubic MgO (Figure 1A).

#### 3.1.2. Fourier Transform Infra-red Spectroscopy (FTIR)

The FTIR spectra of the MgO nanowires showed a major peak at 675 cm-1, confirming the presence of Mg-O vibrations, and bands at 1660 cm-1and 2760 cm-1, which correspond to the characteristic asymmetric stretching modes of C = C and CH_2_ groups, respectively. Furthermore, the band at 3180 cm-1 corresponds to the hydrogen bond between the hydroxyl groups of MgO (Figure 1B).

### 3.2. Hormonal assay

#### 3.2.1. Effect of MgO NPs on the male sex hormones

**Figure 2.**
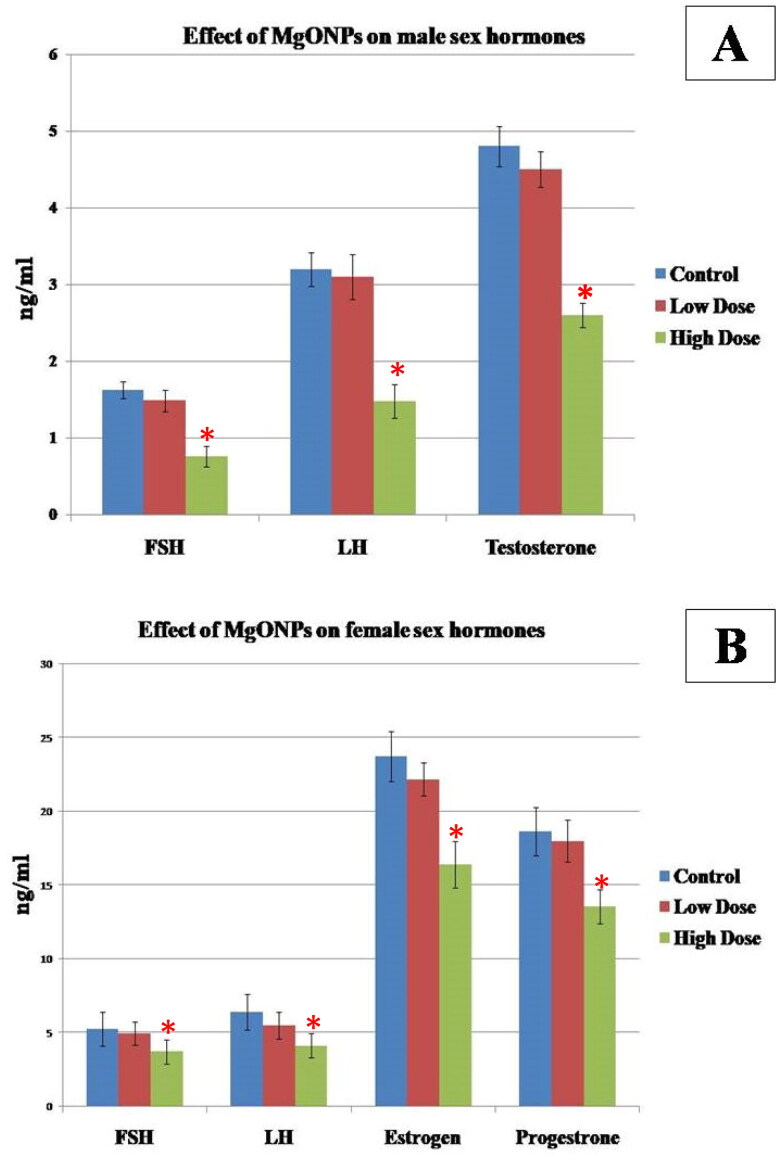
Effect of MgO NPs on A) Serum FSH, LH and testosterone in different groups in male rats. B) Serum FSH, LH, estrogen and progesterone in different groups in female rats. Values were expressed as means ± SD.

The effect of MgO NPs male sex hormones was shown in Figure 2A. There were no statistically significant differences (*p* > 0.05) in serum FSH, LH, and testosterone levels between the low-dose group and the control group. However, in the high-dose group, these hormones significantly (*p* < 0.05) decreased when compared with those in the control group and low-dose group, thus indicating that these hormones were suppressed in a dose-dependent manner.

#### 3.2.2. Effect of MgO NPs on the female sex hormones

The effects of the MgO NPs on the female sex hormones are presented in Figure 2B. Rats in the high dose treated group showed a significant decrease (*p* < 0.05) in FSH, LH, oestrogen (E) and progesterone (P) levels when compared with low dose and control groups. There was no significant difference found between the low dose and control groups (*p* > 0.05).

### 3.3. Oxidative stress and antioxidant enzymes assays

**Figure 3.**
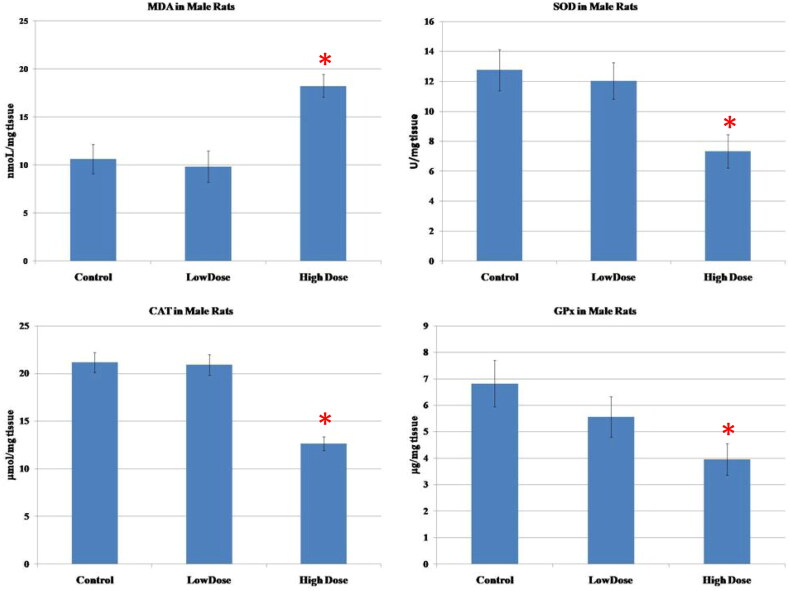
Effect of MgO NPs on the levels of lipid peroxide (MDA) and antioxidative enzymes (SOD, CAT, GPx) in different groups in male rats. Values were expressed as Means ± SD.

The results of oxidative parameters in the testicular homogenate were shown in Figure 3. As seen, the administration of high-dose MgO NPs resulted in a considerable increase in the MDA (marker of lipid peroxidation)as well as a marked diminution of the antioxidant enzymes (SOD, CAT, GPx) in the testicular tissue as compared to low-dose and control groups (*p* < 0.05). Also, there were no significant differences in the level of these parameters between the low-dose group and the control group (*p* > 0.05).

**Figure 4.**
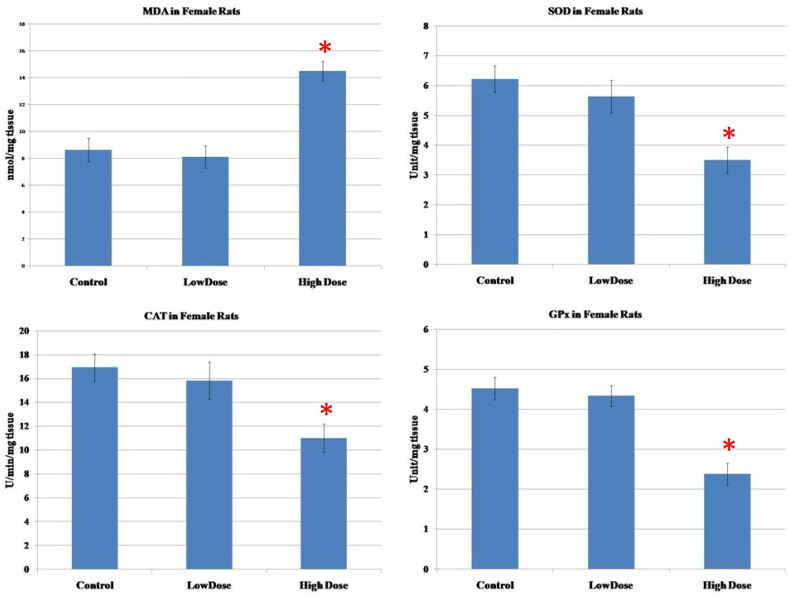
Effect of MgO NPs on the levels of lipid peroxide (MDA) and antioxidative enzymes (SOD, CAT, GPx) in different groups in female rats. Values were expressed as Means ± SD.

The results of oxidative parameters in the ovarian homogenate were shown in Figure 4. As seen, the administration of high dose MgO NPs resulted in a considerable increase in the MDA (marker of lipid peroxidation)as well as a marked diminution of the antioxidant enzymes (SOD, CAT, GPx) in the ovarian tissue as compared to low-dose and control groups (*p* < 0.05). No significant differences in the levels of these parameters were observed in the group treated with a low dose of MgO NPs when compared to the control group (*p* > 0.05).

### 3.4. Histological results in male rats

**Figure 5.**
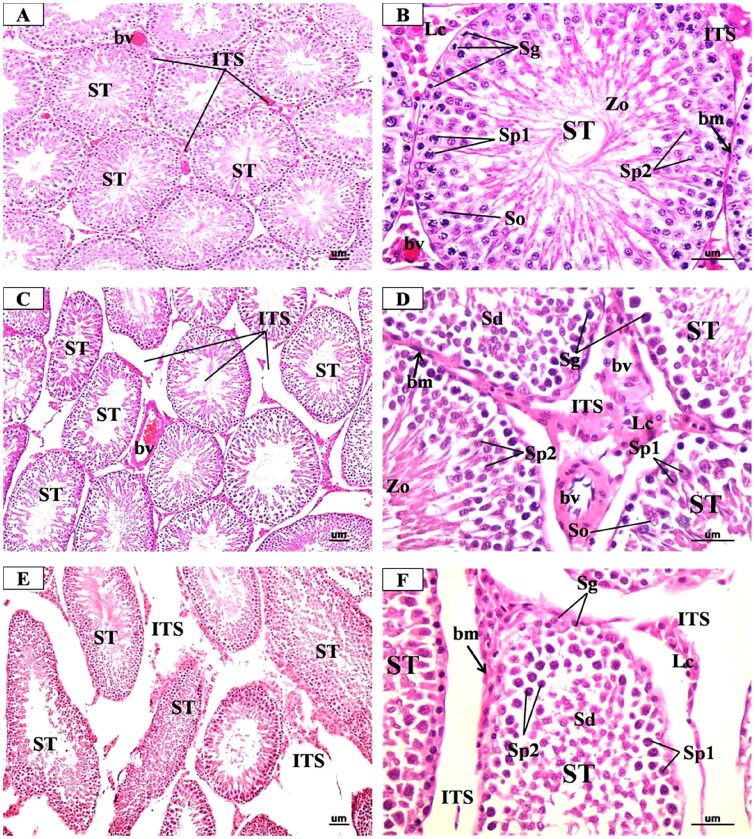
Representative Photomicrographs of H&E-stained transverse sections of the testis from (A & B) Control group showing normal structure of the seminiferous tubules (ST) surrounded by regular thin basement membrane (bm) with spermatozoa (Zo) filled lumina and narrow interstitial spaces (ITS) between the tubules containing little connective tissue and blood vessels (bv). high magnification of ST showed regularly arranged spermatogenic cords formed of spermatogonia (g), primary spermatocytes (Sp1), secondary spermatocytes (Sp2), spermatids (Sd) and Sertoli cells (so). **(C & D)** low dose MgO NPs group showing few changes s compared to the control group where the seminiferous tubules (ST) were slightly separated with the presence of dilated and congested blood vessels (bv). high magnification showed some seminiferous tubules (ST) appeared nearly normal appearance of spermatogenic cells. Other tubules showed little irregularly arranged spermatogenic cords and slight thickening of basement membrane (bm). **(E & F)** High dose MgO NPs group showing disorganised, irregular shapes seminiferous tubules (ST), which appeared atrophic and widely separated with irregular thick basement membrane (bm). high magnification showed degenerated spermatogenic cells and lumina devoid of spermatozoa. The interstitial tissue (is wide and contained few degenerated Leydig cells (Lc). (A, C, E X 200- B, D, F X 400).

The microscopic examination of the testis sections from the control group (Figures 5A & B) revealed the normal structure in the form of well-organized and regular seminiferous tubules separated by a small number of interstitial spaces. The majority of the tubules exhibited thin homogeneous basement membranes and a lining of spermatogenic and Sertoli cells. The spermatogenic cells (spermatogonia, primary and secondary spermatocytes, spermatids, and mature spermatozoa) appeared normal and arranged in an orderly layer from the basement membrane to the lumen of the tubules. Sertoli cells were seen as tall irregular columnar cells with indistinguishable borders between the spermatogenic cells. The interstitial spaces contained little connective tissue, some blood vessels, and clusters of interstitial cells of Leydig with eosinophilic cytoplasm and single eccentric round nucleus.

In the low-dose MgO NPs treated rats (Figures 5C & D), the examination did not show any detectable structural changes in general when compared with the control ones where most seminiferous tubules appeared regular and organized and lined by organized layers of spermatogenic cells with spermatozoa in their lumen and Sertoli cells. However, a few tubules showed thickened basement membrane and slightly degenerated spermatogenic cells. Also, light widening of interstitial spaces with decreased interstitial tissue and decreased number of Leydig cells with few dilated congested blood vessels were also observed. While in the high dose, MgO NPs treated rats (Figures 5E & F), the examination showed more degrees of changes where several seminiferous tubules showed varying degrees of deformation and disarray with some tubules appearing shrunken. The spermatogenic cells in many tubules were degenerated and reduced to a few layers, giving rise to wide lumena as compared to the control. The Sertoli showed fine vacuolations in the basal cytoplasm. The interstitial tissues showed mild edoema, which was evidenced by wide separation of the seminiferous tubules with congestion of blood vessels. The cells of Leydig were reduced in number and their nuclei decreased in size.

### 3.5. Histological results in female rats

**Figure 6.**
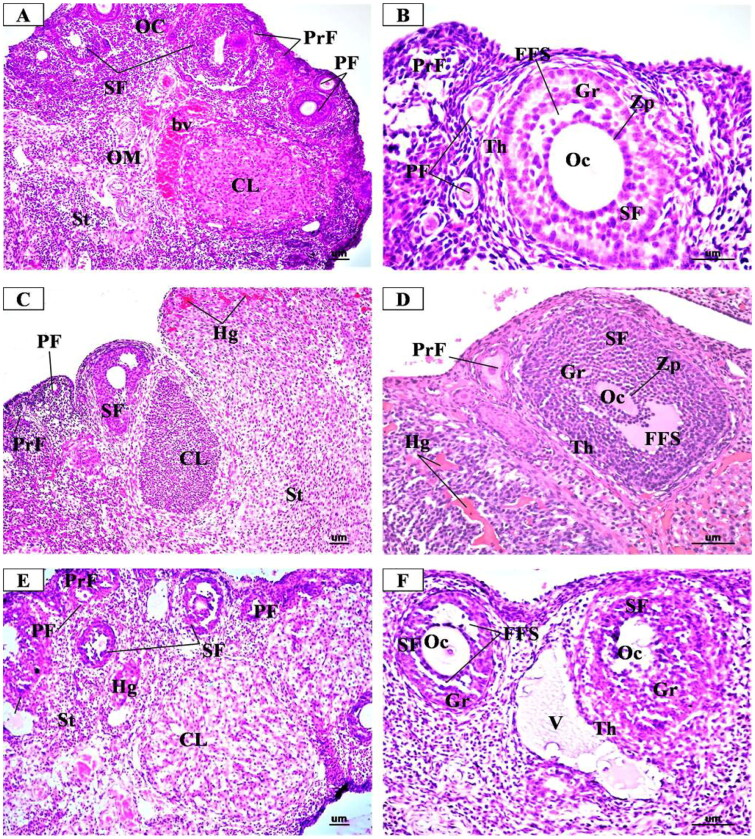
Representative Photomicrographs of H&E stained transverse sections of the ovary from (A & B) Control group showing normal structure of the ovarian cortex (OC) containing follicles at different stages of growth in the form of many primordial follicles (PrF), primary follicles (PF) with oocyte (Oc) and single layer of cuboidal cells, secondary follicle (SF) with large oocyte (Oc) surrounded by clear zona pellucida (Zp), multi-laminar granulosa cells (GC) and theca folliculi (ThF). corpus luteum (CL) is formed of granulosa lutein cells. bc: blood vessel in medulla. AF: atretic follicle. **(C & D)** Low dose MgO NPs treated group showing few changes of the histological structure as compared to the control group with little degenerative changes in the ovarian follicles and increased number of the atretic follicles (AF) and the corpora lutea (CL). some primary follicles (PF) had degenerated granulosa cells and oocytes (Oc). secondary follicles (SF) show atrophic oocytes (Oc) surrounded with degenerated granulosa cells (GC) and many vacuoles (V). dilated and congested blood vessels were observed (BV). some vacuoles are noticed (vv) within the stroma. **(E & F)** High dose MgO NPs treated group showing disturbed ovarian structure with degenerative changes in the ovarian follicles and increased number of the atretic follicles (AF) and the corpora lutea (CL) that showed degenerating luteal cells with pyknotic nuclei and vacuolated cytoplasm (arrow). some primary follicles (PF) had degenerated granulosa cells and oocytes (Oc). secondary follicles (SF) show atrophic oocytes (Oc) surrounded with degenerated granulosa cells (GC) and many vacuoles (V). dilated and congested blood vessels were observed (BV). some vacuoles are noticed (vv) within the stroma. (A, C, E X 200- B, D, F X 400).

The microscopic examination of the ovary sections from the control group (Figures 6A & B), showed the normal structure of the ovarian tissue in the form of an inner medulla and outer cortex that was covered with a single layer of cubical epithelium (germinal epithelium). The medulla was composed of loose connective tissue rich in blood vessels. The cortex contained the ovarian follicles that contain female gametes at different stages of growth and development in the form of primordial, primary, preantral, antral, and mature Graffian follicles as well as corpora lutea and stroma that were composed of collagenous fibres.

In the low dose MgO NPs treated group (Figures 6C&D), the microscopic examination revealed slight changes from the normal histological structure of the ovarian tissue with little damage as compared to the control. This was in the form of a slight decrease in developing ovarian follicles together with presence slight increase of atretic follicles. The ovarian medulla was closely similar to that of the control group despite the presence of some dilated and congested blood vessels.

In the high dose MgO NPs treated group (Figures 6E & F), the microscopic examination revealed more histopathological alterations in the ovarian tissue as compared to the control and low dose group in the form of decreased number of growing follicles, especially the preantral, antral, and Graafian follicles, with increased number of atretic follicles and large corpora lutea. Some preantral follicles had degenerated oocytes, surrounded with vacuolated cuboidal granulosa cells, while some antral follicles showed disorganized and vacuolated granulosa cells with lost cells of corona radiata and cumulus oophorus. In addition, there were dilated and congested blood vessels.

### 3.6. Histomorphometric results

**Figure 7.**
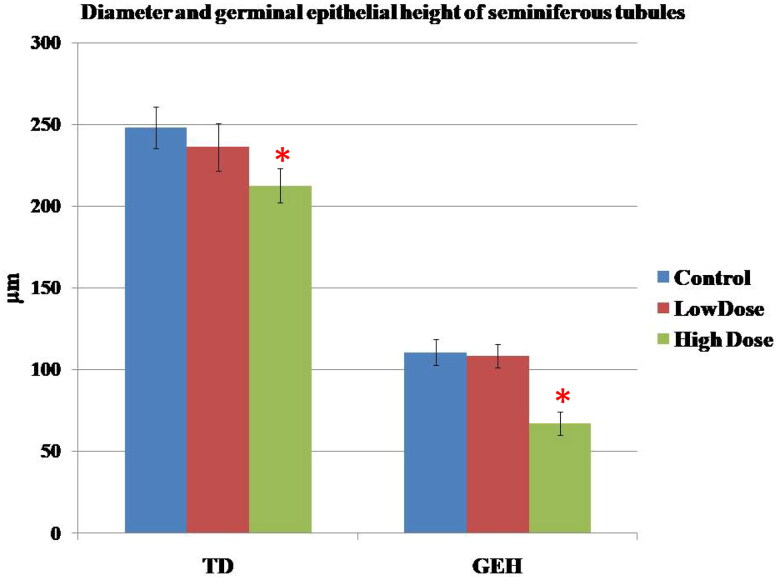
Effect of MgO NPs on the Tubule diameter (TD) and germinal epithelial height (GEH) of seminiferous tubules in different groups in male rats. Values were expressed as means ± SD.

In the seminiferous tubules, the comparisons of the diameter and germinal epithelial height of the tubules in different groups were summarized in Figure 7. Following treatment with high dose MgO NPs, there was a significant decrease in these parameters when compared with those of the control and low dose groups (*p* < 0.05).

**Figure 8.**
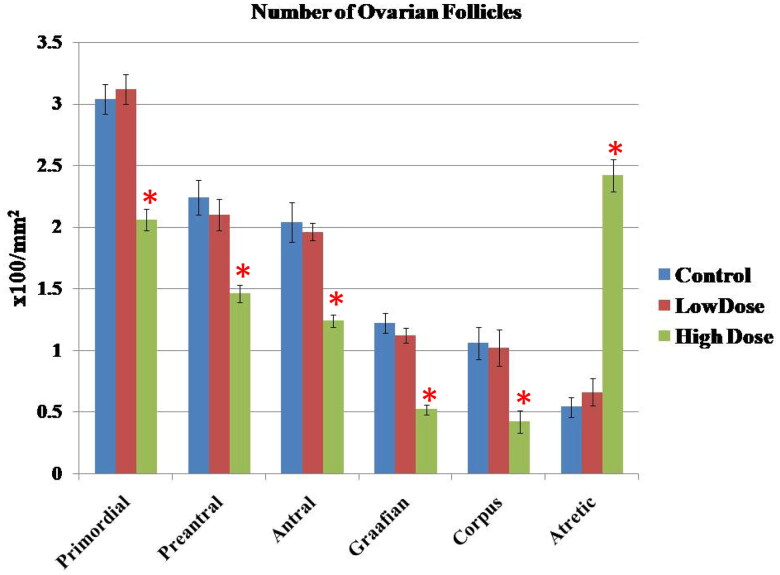
Effect of MgO NPs on the number of various types of the ovarian follicles in different groups in female rats. Values were expressed as Means ± SD.

The number of various types of ovarian follicles in different groups was shown in Figure 8. As seen, high dose of MgO NPs significantly impaired follicular maturation and resulted in reduction of healthy follicles where there was a marked decrease (*p* < 0.05) in the number of all types of follicles with a large number of atretic follicles when compared with the low dose and control groups. On the contrary, low dose of MgO NPs showed non-significant changes (*p* > 0.05) of these follicles as compared with the control group.

## 4. Discussion

MgO NPs have many clinical implications. They demonstrated antibacterial properties against periodontal and cariogenic pathogens such as *Capnocytophagagingivalis, Eikenellacorrodens, Streptococcus sanguinis, Actinomyces israelii*, *Fusobacterium nucleatum*, *Porphyromonasgingivalis*, *Prevotella intermedia*, *Staphylococcus aureus*, *Streptococcus mutans*, and *Streptococcus sobrinus* **[**43]. This was also demonstrated after incorporation into dental material such as dental cements which may aid in resisting periodontal and peri-implant disease and dental caries **[**44,45]. This desired effect may be attributed to enhanced production of reactive oxygen species (ROS), which lead to increased porosity and degradation of cell membranes [44]. However, this may also negatively affect normal cells. The rationale behind this study was to investigate the harmful effects of low versus high doses of MgO NPs on the reproductive organs in males and females where the adverse effects of NPs on the reproductive system are increasingly recognized as an important part of the overall toxicology.

So far, various *in vivo* and *in vitro* models have been developed to study NP-associated toxicity in relative to organs of this system **[**17,46]. Different from other systems, the reproductive system is sexually divided into female and male, which exhibits significant differences. The main function of the male reproductive system is to produce, maintain, and transport sperms, while the female function is to produce the oocytes that are crucial for reproduction as well as to maintain the reproductive function, the reproductive organs, and the glands needed to secrete sex hormones **[**47].

Reviewing the literature, we found that different doses were used in various research works to assess the toxicity of MgO NPs. In the current investigation, two doses of MgO NPs were chosen: low dose (131.5 mg/kg bw equal to 1/10 LD50) versus high dose (263 mg/kg bw equal to 1/5 LD50). These doses were chosen on the basis of a previous study [35] on mice who mentioned that the LD50 for chronic dosage for MgO NPs is 1315 mg/kg of body weight. These authors also mentioned that exposure to a small dose was non-toxic or slightly toxic, but exposure to a high dose was highly toxic to the body. In this respect, the dose‐dependent toxicity of MgO NPs was documented in several organs [48] had shown dose‐dependent pulmonary toxicity in rats in various markers and histopathology of lungs after intra-tracheal instillation of MgO NPs at the doses of 1 mg/kg or 5 mg/kg. Also, [49] had indicated an induced significant DNA damage and chromosomal aberrations after oral administration of three different doses (250, 500, and 1000 mg/kg) of MgO NPs in rats.

The characterization of NPs is required before predicting the toxicity to biological components for understanding the possible toxicological implications **[**]. Hence, in the current study, the MgO NPs were characterized using X-ray diffraction (XRD) and Fourier Transform Infra-red Spectroscopy (FTIR). It was documented that some factors affect the toxicity of NPs such as size, shape, chemical composition, surface charge, solubility, the type of NPs entry path into the body, and the exposure time of NPs. As the size of the particles decreases, the reactivity increases and normally harmless substances may cause hazardous effects and harmful effects may be intensified and vice-versa **[**51,52]. In this study, the crystallographic structure of MgO-NPs was studied using XRD analysis. XRD spectra showed major intense peaks, which matched well with the crystallographic structure according to the JCPDS standard (JCPDS) **[**53]. According to XRD spectra, oxides represented by Mg (OH)_2_ and MgO existed in the synthesized sample **(Nguyen** et al. **2021)**.

In this study, the toxicity of MgO NPs was investigated through the oral route. Although it was documented that NPs are able to get entry into the body *via* several routes including inhalation and dermal and oral routes **[**54], the gastrointestinal route remains to be the main entry and absorption pathways for many NPs that are used in food and drugs, and are distributed to various organs e.g. the liver, kidney, brain and other organs **[**55,56].

The present study has demonstrated significant changes in hormone levels in a dose-dependent manner in both male and female rats in comparison to the control group. In male rats, the current study’s findings demonstrated a decrease in the levels of testosterone, FSH, and LH, which was more pronounced in the groups receiving high dose of MgO NPs than in the low dose and control groups. In accordance, the application of some other NPs produced comparable outcomes where it was reported that the serum levels of testosterone, FSH, and LH dramatically decreased after treatment with PbSe-NP **[**57]. Additionally, [58] found that testosterone, FSH, and LH levels significantly decreased in rats treated with Fe_2_O_3_NPs, Ag NPs, or both compared to the control group, with the combination having the strongest effects. It was reported that this decrease in testosterone might result from testis injury, and the negative feedback effect might be related to a rise in LH and FSH levels. Alternatively, the NPs may affect the hypothalamus and pituitary, resulting in a decrease in LH and FSH secretion, which would then reduce testosterone levels **[**17].

In female rats, our results showed a considerable reduction in the levels of oestrogen, progesterone, FSH, and LH, which was more pronounced in the groups receiving high doses of MgO NPs than in the low-dose and control groups. Some previous investigations using different doses of TiO_2_ NPs **[**59] and MoO3 NPs **[**60] have reported similar results in the reduction of the hormones. Moreover, [61] discovered that varying amounts of Ag NPs administered intraperitoneally for different periods presented a nonsignificant increase in serum progesterone. It was stated that the variation in hormonal levels could be attributed to the direct effect of NPs on the ovaries, which slowed follicular growth and decreased oestrogen, or interfered with follicle function **[**62].

It was reported in several previous studies that some mechanisms, such as oxidative stress, inflammation, and genotoxicity play a role in NPs toxicity and their negative effects on the reproductive system **[**63]. Additionally, it was stated that NPs can infiltrate cells and disrupt their biological structures and activities by increasing intracellular oxidative stress **[**17]. It was documented that many NPs can pass across the blood-testis barrier (BTB), placental barrier, and epithelial barrier, and accumulate in reproductive organs (testis, epididymis, ovary, and uterus); inducing oxidative stress locally, which results in reproductive organ damage that affects sperm quality, quantity, morphology, and motility, as well as reducing the number of mature oocytes and disrupting primary and secondary follicular development **[**64–66].

In this study, the investigation of the oxidative stress markers in testis and ovary homogenates was performed in order to verify the mechanism of toxicity of MgO NPs. Our results showed an increase of MDA, a product of lipid peroxidation, and the reduction of antioxidant enzymes SOD, CAT, and GPx in both testicular and ovarian tissues, which was more significant in the groups receiving high dose of MgO NPs than in the low dose and control groups. One study that conducted inhalation exposure of MgO NPs on rats reported heightened levels of MDA along with a reduction of SOD, catalase, and total antioxidant status, which strongly correlated to ROS production resulting in oxidative stress **[**67]. Furthermore, [68] observed that MgO NPs showed oxidative damage and cytotoxicity through decreased cell metabolic activity in different cell lines. It was also found that MgO NPs can disturb liver cells by producing oxidative stress within the cells, which is associated with hepatotoxicity, further affecting all the liver functions **[**69].

In this study, our findings showed an increased level of MDA; an indicator of lipid peroxidation together with decreased antioxidant enzyme activities, which was eventually accompanied by detrimental effects on both male and female sex hormones. It was documented that oxidative stress has a negative effect on male and female sex hormones as it causes oxidative damage to reproductive cells and intracellular components **[**60,70,71]. Also, the increased oxidative stress affects a variety of feminine physiological processes, including egg maturation, the normal cycling of the ovaries, and follicular development **[**72,73].

The microscopic examination of the current study showed many degenerative changes in both testicular and ovarian tissues, which was more marked in the group receiving high dose of MgO NPs than in the low dose and control groups. In the testis, the high-dose MgO NPs caused many changes as disorganization of seminiferous tubules and depletion of pyknosis of spermatogenic cells as compared to those of the low-dose MgO NPs and control groups. In this regard, it was mentioned that some metal oxide NPs could cause variable degrees of testicular damage, especially with high doses where it was reported that some testicular lesions such as atrophy and increased number of degenerated and disorganized seminiferous tubules occurred upon administration of different doses of zinc oxide (ZnO) NPs in rats **[**74]. Furthermore, it was reported that titanium dioxide (TiO2 NPs) had adverse effects on the testis in mice as they reduced the sperm motility and number of normal sperm but increased the number of abnormal sperm and induction of apoptosis in germ cells **[**75]. In another study, the intraperitoneal injection of Silver NPs (AgNPs) negatively affected the structure of adult rat testes, with the adverse effects increasingly manifesting with an elevated dose and duration of exposure. This effect is demonstrated as disorganized and shrunken seminiferous tubules and detached germinal epithelium, marked reduction in the germinal epithelium, and the absence of sperms **[**76]. Additionally, it was reported that repeated intraperitoneally administration of iron oxide (Fe_2_O_3_) NPs(25 and 50 mg/kg) to mice could be toxic to mice testis with effects such as vacuolization, detachment, and sloughing of germ cells occurring **[**77].

The histometric results of the seminiferous tubules supported the histological findings where there was a statistically significant decrease in the mean values of the height of the germinal epithelial layer and of the perimeter of seminiferous tubules in the tested groups when compared with control groups. In a study by [78], a significant decrease in the diameter of the seminiferous tubules and height of the seminiferous epithelium was found in the animals of the groups that received 50 and 300 mg/kg ZnONPs/day for 35 days when compared with the control group.

Regarding the ovary, the histopathological examination showed many degenerative changes, which were more marked in the group receiving high dose of MgO NPs than in the low dose and control groups. There was a reduced number of growing follicles with the presence of many atretic follicles. Some preantral and antral follicles showed many degenerative changes as degenerated ova and increased apoptotic granulosa cells with vacuolated spaces between them. It was reported that these observed changes in ovarian tissue could be due to diminished gonadotrophin combined with lower oestrogen **[**79]. These results matched those of previous studies using other metal oxide NPs where it was reported that TiO_2_ NPs lead to damage of follicles by reducing follicular survival and preventing the development and maturation of oocytes **[**80]. Also, it was suggested that different doses of silver NPs showed toxic effects on the ovary in the form of inflammation, congestion, and fibrosis witha decreased number of primary and secondary follicles **[**81]. A study by [82] found that rats given high-dose copper (Cu) NPs demonstrated significant ovarian histopathological changes, including ovarian atrophy, disruption of follicular growth, follicular atresia, and a reduction in mature follicles compared to the control group. In addition, a recent study found ovarian tissue alterations in female rats injected with moderate and high doses of ZnO NPs in the form of inflammatory cell infiltration, fibrosis, and follicular cysts **[**83].

The histometric results of the various types of ovarian follicles confirmed the histopathological findings where there was a decrease in the number of preantral, antral, and Graffian follicles, and an increase in the number of atretic follicles. These changes were more significant in the high-dose MgO NPs compared to the low-dose and control groups. These findings matched those of a study of [83], who found similar results in female rats injected with moderate and high doses with ZnO NPs. Furthermore, [82] found that rats given high-dose copper (Cu) NPs demonstrated significant ovarian histopathological changes and a reduction in mature follicles compared to the control group.

## 5. Conclusion

In conclusion, the results from the present study showed that the high dose of MgO NPs displayed both functional and histopathological structural alterations of both testis and ovary in contrast to its low dose, which could be safe for desired applications. Therefore, caution should be exercised in the application of MgO NPs regarding the dose to avoid reproductive complications.

## Data Availability

Data is available from the corresponding author upon reasonable request.

## References

[1] Khan I, Saeed K, Khan I. Nanoparticles: properties, ­applications and toxicities. Arabian J Chem. 2019;12(7):1–15. doi: 10.1016/j.arabjc.2017.05.011.

[2] Kim D, Shin K, Kwon SG, et al. Synthesis and biomedical applications of multifunctional nanoparticles. Adv Mater. 2018;30(49):e1802309. doi: 10.1002/adma.201802309. 30133009

[3] Singh RP. Ch 14 Application of nanomaterials towards development of nanobiosensors and their utility in agriculture. In: Prasad R; Manoj K; Kumar V, editors. “Nanotechnology: an agricultural paradigm” New York, USA: Springer Publisher; 2017, pp 293–303.

[4] Duarah S, Pujari K, Durai RD, et al. Nanotechnology based cosmeceuticals: a review. Int J Appl Pharm. 2016;8:8–12.

[5] Iavicoli I, Leso V, Beezhold DH, et al. Nanotechnology in agriculture: opportunities, toxicological implications, and occupational risks. Toxicol Appl Pharmacol. 2017;329:96–111. doi: 10.1016/j.taap.2017.05.025. 28554660 PMC6380358

[6] Naresh V, Lee N. A review on biosensors and recent development of nanostructured materials-enabled biosensors. Sensors. 2021;21(4):1109. doi: 10.3390/s21041109. 33562639 PMC7915135

[7] Cheng Z, Li M, Raja Dey R, et al. Nanomaterials for cancer therapy: current progress and perspectives. J Hematol Oncol. 2021;14(1):85. doi: 10.1186/s13045-021-01096-0. 34059100 PMC8165984

[8] Yudaev P, Chuev V, Klyukin B, et al. Polymeric dental nanomaterials: antimicrobial action. Polymers. 2022;14(5):864. doi: 10.3390/polym14050864. 35267686 PMC8912874

[9] Mitchell MJ, Billingsley MM, Haley RM, et al. Engineering precision nanoparticles for drug delivery. Nat Rev Drug Discov. 2021;20(2):101–124. – doi: 10.1038/s41573-020-0090-8. 33277608 PMC7717100

[10] Khan M, Khan MSA, Borah KK, et al. The potential exposure and hazards of metal-based nanoparticles on plants and environment, with special emphasis on ZnO NPs, TiO_2_ NPs, and AgNPs: a review. Environ Adv. 2021;6:100128. doi: 10.1016/j.envadv.2021.100128.

[11] Asare N, Instanes C, Sandberg WJ, et al. Cytotoxic and genotoxic effects of silver nanoparticles in testicular cells. Toxicology. 2012;291(1-3):65–72. doi: 10.1016/j.tox.2011.10.022. 22085606

[12] Singh SP, Kumari M, Kumari SI, et al. Genotoxicity of nano-and micron-sized manganese oxide in rats after acute oral treatment. Mutat Res. 2013;754(1–2):39–50. doi: 10.1016/j.mrgentox.2013.04.003. 23618923

[13] Singh SP, Kumari M, Kumari SI, et al. Toxicity assessment of manganese oxide micro and nanoparticles in Wistar rats after 28 days of repeated oral exposure. J Appl Toxicol. 2013b;33(10):1165–1179. doi: 10.1002/jat.2887. 23702825

[14] Singh SP, Rahman MF, Murty USN, et al. Comparative study of genotoxicity and tissue distribution of nano and micron sized iron oxide in rats after acute oral treatment. Toxicol Appl Pharmacol. 2013c;266(1):56–66. doi: 10.1016/j.taap.2012.10.016. 23142030

[15] Liu Y, Li H, Xiao K. Distribution and biological effects of nanoparticles in the reproductive system. Curr Drug Metab. 2016;17(5):478–496. doi: 10.2174/1389200217666160105111436. 26728263

[16] Ema M, Okuda H, Gamo M, et al. A review of reproductive and developmental toxicity of silver nanoparticles in laboratory animals. Reprod Toxicol. 2017;67:149–164. doi: 10.1016/j.reprotox.2017.01.005. 28088501

[17] Wang R, Song S, Wu J, et al. Potential adverse effects of nanoparticles on the reproductive system. Int J Nanomedicine. 2018;13:8487–8506. doi: 10.2147/IJN.S170723. 30587973 PMC6294055

[18] Abdollahii S, Jadidi F, Safari M, et al. Adverse effects of some of the most widely used metal nanoparticles on the reproductive system. J Infertil Reprod Biol. 2020;8(3):22–32.

[19] Limongi T, 2022). Printed Edition of the Special Issue Published in Materials. Ed. Tania Limongi, DISAT Politecnico di Torino Corso DucaDegli Abruzzi 24, Italy.

[20] Nikolova MP, Chavali MS. Metal oxide nanoparticles as biomedical materials. Biomimetics. 2020;5(2):27. doi: 10.3390/biomimetics5020027. 32521669 PMC7345077

[21] Al-Musawi MMS, Al-Shmgani H, Al-Bairuty GA. Histopathological and biochemical comparative study of copper oxide nanoparticles and copper sulphate toxicity in male albino mice reproductive system. Int J Biomater. 2022;2022:4877637–4877612. doi: 10.1155/2022/4877637. 35615428 PMC9126719

[22] Vassal M, Rebelo S, de L, et al. Metal oxide nanoparticles: evidence of adverse effects on the male reproductive system. Int J Mol Sci. 2021;22(15):8061. doi: 10.3390/ijms22158061. 34360825 PMC8348343

[23] Fernandes M, Rb Singh K, Sarkar T, et al. Recent applications of magnesium oxide (MgO) nanoparticles in various domains. Adv Mater Lett. 2020;11(8):1–10. doi: 10.5185/amlett.2020.081543.

[24] Krishnamoorthy K, Manivannan G, Kim SJ, et al. Antibacterial activity of MgO nanoparticles based on lipid peroxidation by oxygen vacancy. J Nanopart Res. 2012;14(9):1063. doi: 10.1007/s11051-012-1063-6.

[25] Krishnamoorthy K, Moon JY, Hyun HB, et al. Mechanistic investigation on the toxicity of MgO nanoparticles toward cancer cells. J Mater Chem. 2012;22(47):24610–24617. doi: 10.1039/c2jm35087d.

[26] Jeevanandam J, Chan YS, Danquah MK. Evaluating the antibacterial activity of MgO nanoparticles synthesized from aqueous leaf extract. Med. One. 2019;4:e190011.

[27] Naguib G, Hassan A, Al-Hazmi F, et al.. Zein based magnesium oxide nanowires: effect of anionic charge on size, release and stability. Digest J nanomater biostruct. 2017;12. 741–749.

[28] Naguib GH, Hosny KM, Hassan AH, et al. Zein based magnesium oxide nanoparticles: assessment of antimicrobial activity for dental implications. Paki J Pharm Sci. 2018;31(1)(Suppl):245–250. 29386150

[29] Naguib GH, Nassar HM, Hamed MT. Antimicrobial properties of dental cements modified with zein-coated magnesium oxide nanoparticles. Bioact Mater. 2022;8:49–56. doi: 10.1016/j.bioactmat.2021.06.011. 34541386 PMC8424389

[30] Di D, He Z, Sun Z, et al. A new nano-cryosurgical modality for tumor treatment using biodegradable MgO nanoparticles. Nanomedicine. 2012;8(8):1233–1241. doi: 10.1016/j.nano.2012.02.010. 22406189

[31] Wahab R, Ansari SG, Dar MA, et al. “Synthesis of magnesium oxide nanoparticles by sol-gel process, In Materials Science Forum (Vol. 558), Trans Tech Publications Ltd.,” 2007, 983–986. doi: 10.4028/www.scientific.net/MSF.558-559.983.

[32] Alaparthi KK, Rajendra Kumar K, Sreedhar Bojja S, et al. A facile and eco-friendly approach for synthesis of magnesium oxide nanoparticles and their antibacterial activity. J Anal Sci Technol. 2018;9(1):21.

[33] Sahayaraj S, Shanmugavel S, Gopinath P, et al. X-ray diffraction investigation of nano-crystalline magnesium oxide (MgO) nanoparticles. J Appl Crystallogr. 2010;43(1):159–163.

[34] Ghosh S, Patil S, Ahire M, et al. Fourier transform infrared spectroscopy analysis of magnesium oxide nanoparticles. Curr Sci. 2011;100(2):173–177.

[35] Shaikh SM, Shyama SK, Prakash V, et al. Absorption, LD50 and effects of CoO, MgO and PbO nanoparticles on mice “Mus musculus. IOSR J Environ Sci, ToxicolFood Technol. 2015;9(2):32–38.

[36] Ohkawa H, Ohishi N, Yagi K. Assay for lipid peroxidase in animal tissue by thiobarbituric acid reaction. Anal Biochem. 1979;95(2):351–358. doi: 10.1016/0003-2697(79)90738-3. 36810

[37] Misra HP, Fridovich I. The role of superoxide anions in the autoxidation of epinephrine and a simple assay for superoxide dismutase. J Biol Chem. 1972;247(10):3170–3175. doi: 10.1016/S0021-9258(19)45228-9. 4623845

[38] Aebi H. Catalase in vitro. Methods enzymol. 1984;105:121–126. doi: 10.1016/s0076-6879(84)05016-3. 6727660

[39] Lawrence RA, Burk RF. Glutathione peroxidase activity in selenium-deficient rat liver. Biochem Biophys Res Commun. 1976;71(4):952–958. doi: 10.1016/0006-291x(76)90747-6. 971321

[40] Bancroft JD, Layton C.. Ch. 10 and 11 The hematoxylin and eosin. In: Suvarna, S.K., Layton, C. and Bancroft, J.D., edsitors. Theory & practice of histological techniques. 7th Edition, Philadelphia: Churchill Livingstone of El Sevier; 2013, 172–214

[41] Mehraein F, Negahdar F. Morphometric evaluation of seminiferous tubules in aged mice testes after melatonin administration. Cell J. 2011;13(1):1–4. 23671820 PMC3652534

[42] Myers M, Britt KL, Wreford NGM, et al. Methods for quantifying follicular numbers within the mouse ovary. Reproduction. 2004;127(5):569–580. doi: 10.1530/rep.1.00095. 15129012

[43] Rodríguez-Hernández AP, Vega-Jiménez AL, Vázquez-Olmos AR, et al. Antibacterial properties in vitro of magnesium oxide nanoparticles for dental applications. Nanomaterials. 2023;13(3):502. doi: 10.3390/nano13030502. 36770464 PMC9921384

[44] Naguib GH, Abd El-Aziz GS, Mously HA, et al. Assessment of the dose-dependent biochemical and cytotoxicity of zein-coated MgO nanowires in male and female albino rats. Ann Med. 2021;53(1):1850–1862. doi: 10.1080/07853890.2021.1991587. 34693843 PMC8547828

[45] Noori AJ, Kareem FA. The effect of magnesium oxide nanoparticles on the antibacterial and antibiofilm properties of glass-ionomer cement. Heliyon. 2019;5(10):e02568. doi: 10.1016/j.heliyon.2019.e02568. 31667407 PMC6812241

[46] Ren L, Zhang J, Zou Y, et al. Silica nanoparticles induce reversible damage of spermatogenic cells via RIPK1 signal pathways in C57 mice. Int J Nanomedicine. 2016;11:2251–2264. doi: 10.2147/IJN.S102268. 27307728 PMC4887058

[47] Hashem AM, Al-Mukhtar S, Ibrahim RH, et al. Anatomy & Physiology of the Reproductive System. https://www.researchgate.net/publication/355475196., October 2021

[48] Gelli K, Porika M, Anreddy R. Assessment of pulmonary toxicity of MgO nanoparticles in rats. Environ Toxicol. 2015;30(3):308–314. doi: 10.1002/tox.21908. 24096598

[49] Mangalampalli B, Dumala N, Perumalla Venkata R, et al. Genotoxicity, biochemical, and biodistribution studies of magnesium oxide nano and microparticles in albino wistar rats after 28-day repeated oral exposure. Environ Toxicol. 2018;33(4):396–410. doi: 10.1002/tox.22526. 29282847

[50] Braakhuis HM, Park MVDZ, Gosens I, et al. Physicochemical characteristics of nanomaterials that affect pulmonary inflammation. Part Fibre Toxicol. 2014;11(1):18. doi: 10.1186/1743-8977-11-18. 24725891 PMC3996135

[51] Almontasser A, Parveen A, A Azam A. Synthesis, characterization and antibacterial activity of magnesium oxide (MgO) nanoparticles. IOP Conf Ser: mater Sci Eng. 2019;577(1):012051. doi: 10.1088/1757-899X/577/1/012051.

[52] Trbojevich RA, Torres AM. Biological synthesis, pharmacokinetics, and toxicity of different metal nanoparticles. Metal Nanoparticles in Pharma. New York: Springer; 2017. p. 451–468.

[53] Abdallah Y, Ogunyemi SO, Abdelazez A, et al. The green synthesis of MgO nano-flowers using rosmarinus officinalis L. (rosemary) and the antibacterial activities against Xanthomonas oryzae pv. oryzae. Biomed Res Int. 2019;2019:5620989. doi: 10.1155/2019/5620989. 30906776 PMC6398066

[54] Yah CS, Simate GS, Iyuke SE. Nanoparticles toxicity and their routes of exposures. Pak J Pharm Sci. 2012;25(2):477–491. 22459480

[55] Geraets L, Oomen AG, Krystek P, et al. Tissue distribution and elimination after oral and intravenous administration of different titanium dioxide nanoparticles in rats. Part Fibre Toxicol. 2014;11(1):30. doi: 10.1186/1743-8977-11-30. 24993397 PMC4105399

[56] Hadrup N, Lam HR. Oral toxicity of silver ions, silver nanoparticles and colloidal silver–a review. Regul Toxicol Pharmacol. 2014;68(1):1–7. doi: 10.1016/j.yrtph.2013.11.002. 24231525

[57] Zhou Q, Yue Z, Li Q, et al. Exposure to PbSe nanoparticles and male reproductive damage in a rat model. Environ Sci Technol. 2019;53(22):13408–13416. doi: 10.1021/acs.est.9b03581. 31362495

[58] Younus AI, Yousef MI, Kamel MA, et al. Changes in semen characteristics and sex hormones of rats treated with iron oxide nanoparticles, silver nanoparticles and their mixture. GSC Biol Pharm Sci. 2020;12(02):229–237.

[59] Gao G, Ze Y, Li B, et al. Ovarian dysfunction and gene-expressed characteristics of female mice caused by long-term exposure to titanium dioxide nanoparticles. J Hazard Mater. 2012;243:19–27. doi: 10.1016/j.jhazmat.2012.08.049. 23131501

[60] Asadi N, Bahmani M, Kheradmand A, et al. The impact of oxidative stress on testicular function and the role of antioxidants in improving it: a review. J Clin Diagn Res. 2017;11(5):IE01–IE05. 28658802 10.7860/JCDR/2017/23927.9886 PMC5483704

[61] Qassim HA, Luaibi N. Study of the hormonal and histological efects of silver nanoparticles on thyroid, ovary and mammary glands in female rats. Reasearch. 2017;118.

[62] Asadi F, Sadeghzadeh M, Jalilvand A, et al. Effect of molybdenum trioxide nanoparticles on ovary function in female rats. J Adv Med Biomed Res. 2019;27(121):48–53. doi: 10.30699/jambs.27.121.48.

[63] Habas K, Demir E, Guo C, et al. Toxicity mechanisms of nanoparticles in the male reproductive system. Drug Metab Rev. 2021;53(4):604–617. doi: 10.1080/03602532.2021.1917597. 33989097

[64] Gao G, Ze Y, Zhao X, et al. Titanium dioxide nanoparticle-induced testicular damage, spermatogenesis suppression, and gene expression alterations in male mice. J Hazard Mater. 2013;258-259:133–143. doi: 10.1016/j.jhazmat.2013.04.046. 23721730

[65] Manke A, Wang L, Rojanasakul Y. Mechanisms of nanoparticle-induced oxidative stress and toxicity. Biomed Res Int. 2013;2013:942916–942915. doi: 10.1155/2013/942916. 24027766 PMC3762079

[66] Muoth C, Aengenheister L, Kucki M, et al. Nanoparticle transport across the placental barrier: pushing the field forward. Nanomedicine. 2016;11(8):941–957. doi: 10.2217/nnm-2015-0012. 26979802

[67] Kiranmai G, Reddy AR. Antioxidant status in MgO nanoparticle-exposed rats. Toxicol Ind Health. 2013;29(10):897–903. doi: 10.1177/0748233712446723. 22673104

[68] Mahmoud A, Ezgi O, Merve A, et al. In vitro toxicological assessment of magnesium oxide nanoparticle exposure in several mammalian cell types. Int J Toxicol. 2016;35(4):429–437. doi: 10.1177/1091581816648624. 27177543

[69] Mekky G, Seeds M, Diab AE, et al. The potential toxic effects of magnesium oxide nanoparticles and valproate on liver tissue. J Biochem Mol Toxicol. 2021;35(3):22676. 10.1002/jbt.22676 33315275

[70] Asadi F, Fazelipour S, Abbasi RH, et al. Assessment of ovarian follicles and serum reproductive hormones in molybdenum trioxide nanoparticles treated rats. Int J Morphol. 2017;35(4):1473–1481. doi: 10.4067/S0717-95022017000401473.

[71] Dutta S, Sengupta P, Slama P, et al. Oxidative stress, testicular inflammatory pathways, and male reproduction. Int J Mol Sci. 2021;22(18):10043. doi: 10.3390/ijms221810043. 34576205 PMC8471715

[72] Agarwal A, Aponte-Mellado A, Premkumar BJ, et al. The effects of oxidative stress on female reproduction: a review. Reprod Biol Endocrinol. 2012;10(1):49. doi: 10.1186/1477-7827-10-49. 22748101 PMC3527168

[73] Kapoor U, Srivastava MK, Srivastava LP. Toxicological impact of technical imidacloprid on ovarian morphology, hormones and antioxidant enzymes in female rats. Food Chem Toxicol. 2011;49(12):3086–3089. doi: 10.1016/j.fct.2011.09.009. 21946071

[74] Mozaffari Z, Parivar K, Roodbari NH, et al. Histopathological evaluation of the toxic effects of zinc oxide (ZnO) nanoparticles on testicular tissue of NMRI adult mice. Asb. 2015;7:275–291. doi: 10.12988/asb.2015.5425.

[75] Alaee S, Ilani M. Effect of titanium dioxide nanoparticles on male and female reproductive systems. JAMSAT. 2017;3(1):3–8. doi: 10.18869/nrip.jamsat.3.1.3.

[76] Ahmed SM, Abdelrahman SA, Shalaby SM. Evaluating the effect of silver nanoparticles on testes of adult albino rats (histological, immunohistochemical and biochemical study). J Mol Histol. 2017;48(1):9–27. doi: 10.1007/s10735-016-9701-4. 27803997

[77] Sundarraj K, Manickam V, Raghunath A, et al. Repeated exposure to iron oxide nanoparticles causes testicular toxicity in mice. Environ Toxicol. 2017;32(2):594–608. doi: 10.1002/tox.22262. 26991130

[78] Talebi A R, Khorsandi L, Moridian M. The effect of zinc oxide nanoparticles on mouse spermatogenesis. J Assist Reprod Genet. 2013;30(9):1203–1209. doi: 10.1007/s10815-013-0078-y. 23949131 PMC3800533

[79] Sato J, Nasu M, Tsuchitani M. Comparative histopathology of the estrous or menstrual cycle in laboratory animals. J Toxicol Pathol. 2016;29(3):155–162. doi: 10.1293/tox.2016-0021. 27559240 PMC4963617

[80] Di Virgilio A, Reigosa M, Arnal P, et al. Comparative study of the cytotoxic and genotoxic effects of titanium oxide and aluminium oxide nanoparticles in Chinese hamster ovary (CHOK1) cells. J Hazard Mater. 2010;177(1-3):711–718. doi: 10.1016/j.jhazmat.2009.12.089. 20079968

[81] Elnoury MAH, Azmy OM, Elshal AOI, et al. Study of the effects of silver nanoparticles exposure on the ovary of rats. Life Sci J. 2013;10(2):1887–1894.

[82] Yang J, Hu S, Rao M, et al. Copper nanoparticle-induced ovarian injury, follicular atresia, apoptosis, and gene expression alterations in female rats. Int J Nanomedicine. 2017;12:5959–5971. doi: 10.2147/IJN.S139215. 28860760 PMC5571856

[83] Hosseini SM, Moshrefi AH, Amani R, et al. Subchronic effects of different doses of zinc oxide nanoparticle on reproductive organs of female rats: an experimental study. Int J Reprod Biomed. 2019;17(2):107–118. doi: 10.18502/ijrm.v17i2.3988. 31435586 PMC6693312

